# Sex Differences in the Outcomes of Elderly Patients with Acute Coronary Syndrome

**DOI:** 10.1155/2020/5091490

**Published:** 2020-05-12

**Authors:** Shi Tai, Xuping Li, Hui Yang, Zhaowei Zhu, Liang Tang, Liyao Fu, Xinqun Hu, Zhenfei Fang, Yonghong Guo, Shenghua Zhou

**Affiliations:** ^1^Department of Cardiology, The Second Xiangya Hospital of Central South University, Changsha, China; ^2^Department of Blood Transfusion, The Second Xiangya Hospital of Central South University, Changsha, China; ^3^Department of Geriatrics, The Second Xiangya Hospital of Central South University, Changsha, China

## Abstract

**Background:**

The impact of sex on the outcome of patients with acute coronary syndrome (ACS) has been suggested, but little is known about its impact on elderly patients with ACS.

**Methods:**

This study analyzed the impact of sex on in-hospital and 1-year outcomes of elderly (≥75 years of age) patients with ACS hospitalized in our department between January 2013 and December 2017.

**Results:**

A total of 711 patients were included: 273 (38.4%) women and 438 (61.6%) men. Their age ranged from 75 to 94 years, similar between women and men. Women had more comorbidities (hypertension (79.5% vs. 72.8%, *p*=0.050), diabetes mellitus (35.2% vs. 26.5%, *p*=0.014), and hyperuricemia (39.9% vs. 32.4%, *p*=0.042)) and had a higher prevalence of non-ST-segment elevation ACS (NSTE-ACS) (79.5% vs. 71.2%, *p*=0.014) than men. The prevalence of current smoking (56.5% vs. 5.4%, *p* < 0.001), creatinine levels (124.4 ± 98.6 vs. 89.9 ± 54.1, *p* < 0.001), and revascularization rate (39.7% vs. 30.0%, *p*=0.022) were higher, and troponin TnT and NT-proBNP tended to be higher in men than in women. The in-hospital mortality rate was similar (3.5% vs. 4.4%, *p*=0.693), but the 1-year mortality rate was lower in women than in men (14.7% vs. 21.7%, *p*=0.020). The multivariable analysis showed that female sex was a protective factor for 1-year mortality in all patients (OR = 0.565, 95% CI 0.351–0.908, *p*=0.018) and in patients with STEMI (OR = 0.416, 95% CI 0.184–0.940, *p*=0.035) after adjustment.

**Conclusions:**

Among the elderly patients with ACS, the 1-year mortality rate was lower in women than in men, which could be associated with comorbidities and ACS type.

## 1. Introduction

Cardiovascular disease (CVD) is the leading cause of death for both men and women worldwide [1]. Acute coronary syndrome (ACS), a major clinical manifestation of atherothrombosis, refers to a wide spectrum of clinical presentations, such as ST-segment elevation myocardial infarction (STEMI) and non-ST-segment elevation ACS (NSTE-ACS), and increases with age, and the outcomes of ACS in elderly patients are generally worse than those in young patients [2]. Almost one out of every two patients hospitalized for ACS is over 75 years of age [3, 4], and the in-hospital mortality due to ACS ranges from 4% to10% [4, 5].

Women account for approximately 30% of patients presenting with ACS [4, 6] and have long been described as being “older and sicker” than their male counterparts [7]. In particular, significant sex-related differences exist in ACS presentation, management, and outcomes [7]. A large contemporary registry study in China demonstrated that women hospitalized for ACS received acute treatments and secondary prevention less frequently and had a higher in-hospital mortality than men due to poor clinical profiles and little evidence for acute treatments [4]. To date, little is known about the sex-related differences in elderly patients with ACS.

The Italian Elderly ACS study included patients with NSTE-ACS and ≥75 years of age and showed that women had poor 1-year primary outcomes, including death, nonfatal myocardial infarction, disabling stroke, cardiac rehospitalization, and severe bleeding [8]. According to a nationwide registry study in the Netherlands, the relation between sex and mortality appeared to be age-dependent, with increased mortality in women at a young age and decreased mortality in women at an advanced age [9]. It is still unclear whether these differences can be solely explained by sex or by other factors such as age, extent or impact of risk factors, clinical presentation, and treatment strategies. Therefore, this study focused on patients with ACS and ≥75 years and aimed to investigate the sex differences in the clinical characteristics, in-hospital management, adverse events, and 1-year mortality among those patients.

## 2. Methods

### 2.1. Study Design and Patients

This retrospective single-center study included 711 consecutive patients with ACS and ≥75 years of age who were hospitalized in our department between January 2013 and December 2017. Only patients who were initially admitted to our center were included; those who were transferred from other centers were excluded. The diagnostic criteria for ACS were based on the presence of chest pain or discomfort, electrocardiogram (ECG) findings, and myocardial injury biomarker measurements. The study protocol conformed to the ethical guidelines of the 1975 Declaration of Helsinki and was approved by the Human Research Committee of the Second Xiangya Hospital of Central South University. The need for individual consent was waived by the committee.

### 2.2. Definitions and Endpoints

ACS was defined in accordance with the guidelines published by the American College of Cardiology for the diagnosis and management of patients with ST-segment elevation myocardial infarction (STEMI) and non-ST-segment elevation ACS (NSTE-ACS) [10, 11]. Renal insufficiency (CKD ≥3) was defined as an estimated glomerular filtration rate (eGFR) < 60 mL/min per 1.73 m^2^. In our study, severe heart failure indicated class III-IV heart failure, according to the Killip or New York Heart Association classification system. Readmission was defined as any readmission after discharge. Stroke was defined as the sudden onset of focal neurological deficit resulting from either cerebral infarction or hemorrhage. According to the Bleeding Academic Research Consortium (BARC) criteria, BARC types 2 and 3 were included as in-hospital bleeding events [12]. A BARC type 2 event was defined as clinically overt hemorrhage requiring medical attention, whereas a BARC type 3 event was defined as bleeding, including gastrointestinal bleeding, respiratory bleeding, and genitourinary bleeding, with a hemoglobin decrease of at least 3 g/dl, requiring transfusion or surgical intervention.

The primary outcome was 1-year all-cause mortality. The secondary outcomes included the rates of revascularization, readmission, and stroke over 1 year of follow-up.

### 2.3. Data Collection

The following data were collected: body weight, height, and body mass index (BMI) during hospitalization, diabetes, atrial fibrillation, chronic kidney disease, history of chest pain, history of CVD treatment, smoking, laboratory parameters, length of hospital stay, demographic characteristics, medication, in-hospital management, and adverse events. The following biochemical parameters were also extracted from the medical charts: hemoglobin, total cholesterol, high-density lipoprotein cholesterol (HDL-C), low-density lipoprotein cholesterol (LDL-C), triglycerides, and glycated hemoglobin (HbA1c).

### 2.4. Follow-Up

In-hospital outcomes were ascertained by a hospital chart review. After discharge, participant follow-up was carried out by means of outpatient visits and telephone calls for up to one year.

### 2.5. Statistical Analysis

Categorical variables were presented as numbers (percentages) and compared using the Pearson chi-squared tests or Fisher's exact test. Continuous variables were presented as mean ± SD and compared using Student's *t*-test if the data were normally distributed. Nonnormally distributed continuous variables were presented as medians (interquartile ranges) and compared using nonparametric tests. Odds ratios (ORs) were presented with 95% confidence intervals (CIs). Univariable factor logistic regression was used to analyze the risk factors associated with 1-year all-cause mortality. A multivariable logistic regression analysis was used to define the independent determinants of 1-year all-cause mortality after adjusting for comorbidities, presentation, and clinical profiles. Meaningful factors, defined by the univariable *p* < 0.05, including age (Model 1); hypertension; current smoking; severe heart failure (Model 2); hemoglobin, platelet, total cholesterol, HDL-C, LDL-C, creatinine, and serum uric acid levels (Model 3), were included in the multivariable logistic regression analysis. A two-tailed *p*-value <0.05 indicated statistical significance. All statistical analyses were conducted using SPSS 22.0 (IBM, Armonk, NY, USA).

## 3. Results

### 3.1. Characteristics of the Patients

There were 273 (38.4%) women and 438 (61.6%) men. They were 75–94 years of age, and the age distribution was similar between women and men (78 [76–81] vs. 78 [76–80], *p*=0.381).

### 3.2. Characteristics of the Patients according to Sex

The baseline characteristics of each group are listed in Table 1. Women had a higher prevalence of traditional risk factors for CVD, including hypertension (79.5% vs. 72.8%, *p*=0.050), diabetes mellitus (35.2% vs. 26.5%, *p*=0.014), and hyperuricemia (39.9% vs. 32.4%, *p*=0.042), but a lower prevalence of current smoking (5.4% vs. 56.5%, *p* < 0.001) than men. The prevalence of severe heart failure on admission (56.8% vs. 46.0%, *p*=0.005) and NSTE-ACS (79.5% vs. 71.2%, *p*=0.014) was significantly higher, while STEMI (20.5% vs. 28.8%, *p*=0.014) was less frequent in women than in men. Platelet counts (202.9 ± 64.9 vs. 174.3 ± 61.4, *p* < 0.001), total cholesterol (4.1 ± 1.0 vs. 3.8 ± 0.9, *p* < 0.001), LDL-C (2.4 ± 0.9 vs. 2.2 ± 0.8, *p*=0.002), and HDL-C (1.1 ± 0.3 vs. 1.0 ± 0.3, *p*=0.002) were significantly higher, whereas hemoglobin levels (110.4 ± 18.3 vs. 121.8 ± 20.2, *p* < 0.001), creatinine levels (89.9 ± 54.1 vs. 124.4 ± 98.6, *p* < 0.001), and serum uric acid (349.2 ± 119.1 vs. 381.4 ± 110.1, *p* < 0.001) were significantly lower in women than in men. Regarding in-hospital management, the coronary revascularization rate was significantly lower in women than in men (30.0% vs. 39.7%, *p*=0.022), while the medication rate and rate of intra-aortic balloon pump (IABP) use were similar between the two groups. There were no differences in in-hospital adverse events. A total of 135 patients died (19.0%), and the 1-year mortality rate was significantly lower in women than in men (14.7% vs. 21.7%, *p*=0.020). The percentages of rehospitalization, revascularization, stroke, and bleeding after discharge were similar between the two groups.

### 3.3. Differences between Women and Men according to ACS Type

The results of the subgroup (NSTE-ACS and STEMI) comparisons are shown in Table 2. There were 182 (25.8%) patients with STEMI and 529 (74.2%) with NSTE-ACS. Among the 256 (36%) patients who underwent coronary revascularization, 93 (51.1%) were STEMI patients, and 163 (30.8%) were NSTE-ACS patients. Sex-related differences, including the prevalence of current smoking, and hemoglobin, platelet, total cholesterol, LDL-C, creatinine, and serum uric acid levels were still observed in both STEMI and NSTE-ACS subgroups. In the NSTE-ACS subgroup, the rates of hyperuricemia (41.9% vs. 31.4%, *p*=0.013), severe heart dysfunction on admission (60.8% vs. 47.9%, *p*=0.003), high HDL-C (1.1 ± 0.3 vs. 1.0 ± 0.3, *p*=0.002), and the use of proton pump inhibitors (PPIs) (81.2% vs. 72.6%, *p*=0.024) were significantly higher, while the coronary revascularization rate (22.6% vs. 36.5%, *p*=0.002) was lower in women than in men, but these differences were not observed in the STEMI subgroup. In patients with STEMI, the rate of diabetes mellitus (48.2% vs. 19.8%, *p* < 0.001) was higher in women than in men and 1-year mortality (19.6% vs. 38.1%, *p*=0.014) was lower.

### 3.4. Multivariable Analyses between Women and Men

To evaluate whether the residual sex difference in mortality could be explained by disparities in the risk factors, we examined the independent determinants of 1-year all-cause mortality. As shown in Table 3, female patients had a significantly lower unadjusted risk of death (unadjusted OR = 0.620, 95% CI 0.413–0.929, *p*=0.021) than male patients. The multivariable logistic regression analysis showed similar results (OR = 0.597, 95% CI 0.397–0.900, *p*=0.014) after adjusting for age (Model 1). Additional variables included diabetes mellitus, hypertension, current smoking, and severe heart failure (Model 2) and hemoglobin, platelet, total cholesterol, HDL-C, LDL-C, creatinine, and serum uric acid levels (Model 3). The same associations were observed in Model 2 (OR = 0.531, 95% CI 0.347–0.811, *p*=0.003) and Model 3 (OR = 0.565, 95% CI 0.351–0.908, *p*=0.018). Similar results were observed in elderly patients with STEMI but not in elderly patients with NSTE-ACS.

Regarding the adjustment variables in Model 3, in all patients, age (OR = 1.08, 95% CI: 1.02–1.15, *p*=0.010), severe heart failure (OR = 1.77, 95% CI: 1.14–2.75, *p*=0.011), PLT levels (OR = 1.004, 95% CI: 1.001–1.007, *p*=0.018), and creatinine levels (OR = 1.005, 95% CI: 1.003–1.007, *p* < 0.001) were associated with mortality, along with sex (OR = 0.57, 95% CI: 0.35–0.91, *p*=0.018). In patients with STEMI, severe heart failure (OR = 3.84, 95% CI: 1.89–7.78, *p* < 0.001) was associated with mortality, along with sex (OR = 0.42, 95% CI: 0.18–0.94, *p*=0.035). In patients with NSTE-ACS, age (OR = 1.09, 95% CI: 1.01–1.18, *p*=0.033), hemoglobin (OR = 0.98, 95% CI: 0.97–1.00, *p*=0.027), LDL (OR = 1.49, 95% CI: 1.07–2.08, *p*=0.018), and creatinine (OR = 1.004, 95% CI: 1.002–1.007, *p*=0.001) were associated with mortality.

## 4. Discussion

The main findings of the present study are as follows: (1) elderly women had more comorbidities and were more likely to present with NSTE-ACS than men in the total ACS cohort; (2) in women with NSTE-ACS, the prevalence of severe heart failure on admission was higher, and they underwent coronary revascularization less often than men, but in-hospital adverse events and 1-year mortality were similar between women and men; (3) in STEMI patients, there were no differences in the in-hospital treatments and in-hospital adverse events between women and men, but women had lower 1-year mortality than men; and (4) female sex was a protective factor for 1-year mortality in all populations, especially in patients with STEMI.

Although traditional atherosclerotic disease risk factors are important for both men and women with ACS, some factors accumulated more often in female patients. In the present study, women had higher rates of traditional risk factors, including hypertension, diabetes, hyperuricemia, elevated LDL-C, and severe heart failure, than men. In contrast, men were more likely to be smokers and have elevated creatinine levels. Notably, these differences were similar across both types of ACS (STEMI and NSTE-ACS). These risk factor distribution patterns have been confirmed by other studies in the whole populations [7] and in elderly patients [4, 9]. In addition, women have sex-specific risk factors, such as pregnancy and menopause [7, 13]. It is accepted that the cardiovascular risk profile of women worsens during postmenopause, and the prevalence of coronary artery disease (CAD) steeply increases with age thereafter. We also observed that among the elderly patients with ACS, women were more likely to present with NSTE-ACS than men, whereas men presented with STEMI more often than women. This finding is in agreement with data derived from the Improving Care for Cardiovascular Disease in China (CCC) Project, in that the prevalence of STEMI was significantly lower in women than in men, and women were more likely to present with NSTE-ACS compared to their male counterparts [4]. An increased burden of plaque erosion, coronary vasospasm, spontaneous coronary artery dissection, and stress-related cardiomyopathy in women might partly be related to the observed phenomenon [7].

In terms of in-hospital management among patients with NSTE-ACS, there were no differences between women and men, except for the use of PPIs. We believe that the use of PPIs occurred more often in women than in men, which could be explained by the increased bleeding risk in women with ACS [8, 14, 15]. Nevertheless, no difference was observed regarding in-hospital bleeding in the present study; the use of PPIs might partly contribute to this finding. In the present study, we observed a lower coronary revascularization rate in women hospitalized for NSTE-ACS than in men. This result is in line with data from the CCC project, which showed that eligible women with NSTE-ACS were less likely to undergo timely percutaneous coronary intervention (PCI) than men with NSTE-ACS (30.5% vs. 34.2%, *p* < 0.001) [4]. We believe that the fear of complications associated with invasive treatments might, in part, explain this finding because women, especially older women, might be considered too fragile to undergo aggressive treatments. A surprising finding in our study was the similar in-hospital clinical outcomes between women and men, despite fewer PCI carried out in women. This finding was in contrast with a previous report that showed that women with NSTE-ACS had higher crude in-hospital mortality rates than men with NSTE-ACS [4]. Because our cohort included only patients of ≥75 years of age, the above difference might originate from the different age groups. In addition, a recent report confirmed that women with NSTE-ACS ≥70 years of age had better outcomes than those <70 years [8]. Another study using data from the National Inpatient Sample (NIS) database in the United States indicated that women had lower risk-adjusted in-hospital mortality than men after accounting for differences in age and comorbidities [16]. These findings suggested that the relation between sex and mortality was age-dependent, with increased mortality in women at a young age and decreased mortality in women at an advanced age.

Among the patients with STEMI, there were no differences regarding in-hospital treatments and in-hospital adverse events between women and men, but women had better 1-year outcomes than men in the present study. Some studies demonstrated increased rates of mortality among women, some studies indicated no difference, and other studies showed lower rates of mortality in women than in men [17]. These controversial results may be explained by potential interactions between age and sex; significant differences in in-hospital mortality rates between women and men with STEMI were demonstrated when the cohort was stratified by age groups (<55 years, 55–64 years, and >75 years) [4]. Younger age was associated with higher 30-day mortality rates in women with STEMI, but this difference decreased after age 60 and was no longer observed in elderly women [18]. In fact, mortality in elderly women was lower than that in age-matched men, as shown by previous studies [19, 20]; this result was further confirmed by the Netherlands National Trial Register, which showed that excess mortality in women mostly occurred in young patients with STEMI, while older women had a better outcome than men of the same age [9]. These findings suggest that there is an age-dependent relationship among the outcomes between male and female patients with STEMI. Our results show that elderly female patients with STEMI have lower 1-year mortality than elderly male patients with STEMI.

Previous studies have shown that sex differences in early mortality after ACS could be largely explained by the clinical differences at presentation [21, 22]. To evaluate whether the sex differences associated with 1-year mortality could be explained by disparities in clinical characteristics, we adjusted for comorbidities, presentation, and clinical profiles. After adjusting for age (Model 1); age, diabetes mellitus, hypertension, current smoking, and severe heart failure (Model 2); and hemoglobin, platelet counts, total cholesterol, HDL-C, LDL-C, creatinine, and serum uric acid (Model 3), the female sex was consistently shown as an independent protective factor for 1-year mortality in the whole cohort, especially among patients with STEMI. Nevertheless, a delay in presentation [1, 4, 23] and angiographic severity of coronary lesions [17], which may also contribute to the sex difference in mortality after ACS, were not adjusted in the present study. In addition, a previous study indicated that the more favorable mortality rate in older women could be attributed to the shorter exposure to obstructive coronary disease and longer life expectancy in women than in men [18]. In all patients, sex, age, severe heart failure, PLT, and creatinine were all independently associated with mortality. In patients with STEMI, only sex and severe heart failure were independently associated with mortality. Since all patients were menopausal, the differences cannot be attributed to estrogens, and other factors are also involved in the mortality risk. In patients with NSTE-ACS, age, hemoglobin, LDL, and creatinine were independently associated with mortality, but not sex. Therefore, in NSTE-ACS, other traditional risk factors for mortality play more important roles in the risk of mortality. Nevertheless, the mechanism for the sex disparity in 1-year mortality, especially in elderly patients with STEMI, still needs to be further investigated in future studies.

## 5. Limitations

This study has some limitations. First, this was a single-center experience and included a small number of patients. Second, residual measured and unmeasured confounding factors, including changes in ECG parameters, might have contributed to some of these findings but were not included in the regression model. Third, the data were based on the routine clinical parameters measured in the management of ACS, which do not include sexual hormone levels. Finally, the details of the procedural characteristics, especially the time intervals for STEMI and angiographic severity of coronary lesions, are important in view of the previously described sex differences in the literature, but these factors were not analyzed in this study.

## 6. Conclusion

Our study showed that elderly women with ACS had more comorbidities and were more likely to present with NSTE-ACS than men in the total cohort, similar to other studies. A surprising finding was the better 1-year outcome in elderly women with STEMI than in elderly men with STEMI, while in-hospital and 1-year outcomes were similar between elderly women and men with NSTE-ACS. It is worth noting that the female sex was an independent protective factor for 1-year mortality in the whole ACS cohort, especially in patients with STEMI.

## Figures and Tables

**Table 1:** Differences between women and men.

	Women (*n* = 273)	Men (*n* = 438)	*p*
Demographics and medical history			
Age, yrs (median, IQR)	78 (76–81)	78 (76–80)	0.381
Body mass index (median, IQR)	22.7 (20.6–25.4)	23.1 (20.9–25.4)	0.427
Diabetes mellitus, no. (%)	96 (35.2)	116 (26.5)	0.014
Hypertension, no. (%)	217 (79.5)	319 (72.8)	0.050
Atrial fibrillation, no. (%)	30 (11.2)	53 (12.3)	0.656
CKD ≥ 3	13 (4.8)	26 (5.9)	0.506
Stroke, no. (%)	32 (11.9)	71 (16.5)	0.098
Previous chest pain, no. (%)	181 (67.5)	286 (66.2)	0.716
Previous PCI, no. (%)	36 (13.5)	78 (18.1)	0.112
Previous CABG, no. (%)	3 (1.1)	10 (2.3)	0.255
Current smoking, no. (%)	14 (5.4)	239 (56.5)	<0.001
Clinical presentation			
Hyperuricemia, no. (%)	109 (39.9)	142 (32.4)	0.042
Severe heart failure, no. (%)	155 (56.8)	201 (46.0)	0.005
STEMI, no. (%)	56 (20.5)	126 (28.8)	0.014
NSTE-ACS, no. (%)	217 (79.5)	312 (71.2)	0.014
Laboratory data			
WBC, 10^9^/L (mean ± SD)	7.4 ± 3.3	7.2 ± 3.2	0.568
Hemoglobin, g/L (mean ± SD)	110.4 ± 18.3	121.8 ± 20.2	<0.001
Platelets, 10^9^/L (mean ± SD)	202.9 ± 64.9	174.3 ± 61.4	<0.001
Fasting glucose, mmol/l (median, IQR)	6.0 (4.9–7.6)	5.7 (4.8–7.1)	0.181
HbA1C, % (median, IQR)	6.7 (6.0–7.4)	6.1 (5.6–6.5)	0.005
ALT, u/l (median, IQR)	17.5 (11.7–28.3)	19.9 (13.6–33.5)	0.012
AST, u/l (median, IQR)	22.6 (17.1–39.5)	22.6 (17.8–49.9)	0.579
Albumin, g/L (mean ± SD)	35.1 ± 4.5	35.1 ± 4.1	0.902
Triglycerides, mmol/l (mean ± SD)	1.6 ± 1.0	1.6 ± 4.2	0.960
Total cholesterol, mmol/l (mean ± SD)	4.1 ± 1.0	3.8 ± 0.9	<0.001
HDL-C, mmol/l (mean ± SD)	1.1 ± 0.3	1.0 ± 0.3	0.002
LDL-C, mmol/l (mean ± SD)	2.4 ± 0.9	2.2 ± 0.8	0.002
Creatinine, *μ*mmol/l (mean ± SD)	89.9 ± 54.1	124.4 ± 98.6	<0.001
Serum uric acid, *μ*mmol/l (mean ± SD)	349.2 ± 119.1	381.4 ± 110.1	<0.001
PT, sec (mean ± SD)	13.7 ± 4.3	13.5 ± 2.8	0.674
APTT, sec (mean ± SD)	39.5 ± 14.4	41.4 ± 22.2	0.562
CK-Mb, u/l (mean ± SD)	35.0 ± 71.4	44.3 ± 119.0	0.266
TnT, pg/ml (median, IQR)	19.3 (9.7–603.3)	33.2 (12.3–1461.0)	0.141
hs-CRP, mg/l (median, IQR)	4.3 (1.3–23.2)	5.1 (1.4–20.2)	0.849
NT-proBNP, pg/ml (median, IQR)	935.9 (290.4–3082.5)	1337.0 (394.9–3737.7)	0.067
EF, % (mean ± SD)	53 ± 10.4	53 ± 10.9	0.460
In-hospital management			
Aspirin, no. (%)	227 (83.2)	376 (86.2)	0.262
Clopidogrel, no. (%)	234 (86.0)	391 (89.5)	0.168
ACEI/ARB, no. (%)	168 (61.5)	268 (61.2)	0.925
Beta blocker, no. (%)	206 (75.5)	306 (69.9)	0.106
Statin, no. (%)	265 (97.8)	422 (96.3)	0.283
Diuretic, no. (%)	128 (47.6)	210 (48.6)	0.791
PPI, no. (%)	227 (84.4)	340 (78.7)	0.063
IABP, no. (%)	7 (2.6)	18 (4.2)	0.255
Revascularization	82 (30.0)	174 (39.7)	0.022
In-hospital events			
Heart failure, no. (%)	42 (15.7)	77 (17.9)	0.458
Bleeding, no. (%)	22 (8.2)	39 (9.1)	0.706
Ventricular tachycardia, no. (%)	23 (8.6)	36 (8.3)	0.920
Stroke, no. (%)	5 (1.9)	2 (0.5)	0.071
Death, no. (%)	4 (3.5)	8 (4.4)	0.693
One-year follow-up			
Revascularization	4 (1.6)	7 (1.9)	0.801
Readmission, no. (%)	96 (36.9)	159 (39.9)	0.436
Stroke, no. (%)	13 (5.2)	18 (4.8)	0.827
Death, no. (%)	40 (14.7)	95 (21.7)	0.020

STEMI: ST-segment elevation myocardial infarction; NSTE-ACS: non-ST-elevation acute coronary syndrome; WBC: white blood cell; ALT: glutamic-pyruvic transaminase; AST: glutamic-oxaloacetic transaminase; HDL-C: high-density lipoprotein cholesterol; LDL-C: low-density lipoprotein cholesterol; PT: prothrombin time; APTT: activated partial thromboplastin time; ACEI/ARB: angiotensin-converting enzyme inhibitors/angiotensin receptor blockers; PPI: proton pump inhibitor; IABP: intra-aortic balloon pump; CKD: chronic kidney disease; and CKD ≥ 3: estimated glomerular filtration rate (eGFR) < 60 mL/min per 1.73 m^2^.

**Table 2:** Differences between women and men according to ACS type.

	NSTE-ACS			STEMI		
	Women (*n* = 217)	Men (*n* = 312)	*p*	Women (*n* = 56)	Men (*n* = 126)	*p*
Demographics and medical history						
Age, yrs (median, IQR)	78 (76–81)	78 (76–80)	0.085	77 (76–80)	78 (76–81)	0.218
Body mass index, (median, IQR)	22.7 (20.5–25.4)	23.2 (20.9–25.5)	0.275	22.3 (20.7–25.3)	22.3 (19.8–24.6)	0.703
Diabetes mellitus, no. (%)	69 (31.8)	91 (29.2)	0.517	27 (48.2)	25 (19.8)	<0.001
Hypertension, no. (%)	182 (83.7)	248 (79.5)	0.204	35 (62.5)	71 (56.3)	0.437
Atrial fibrillation, no. (%)	19 (8.9)	35 (11.4)	0.362	11 (19.6)	18 (14.4)	0.374
CKD ≥ 3	9 (4.2)	14 (4.5)	0.853	4 (7.1)	12 (9.5)	0.601
Stroke, no. (%)	25 (11.8)	54 (17.6)	0.068	7 (12.5)	17 (13.7)	0.825
Previous chest pain, no. (%)	148 (69.8)	219 (71.3)	0.708	33 (58.9)	67 (53.6)	0.505
Previous PCI, no. (%)	32 (15.1)	69 (22.5)	0.037	4 (7.3)	9 (7.2)	0.986
Previous CABG, no. (%)	3 (1.4)	10 (3.3)	0.187	0 (0)	0 (0)	NA
Current smoking, no. (%)	7 (3.4)	166 (55.5)	<0.001	7 (12.5)	73 (58.9)	<0.001
Clinical presentation						
Hyperuricemia, no. (%)	91 (41.9)	98 (31.4)	0.013	18 (32.1)	44 (34.9)	0.715
Severe heart failure, no. (%)	132 (60.8)	149 (47.9)	0.003	23 (41.1)	52 (41.3)	0.980
Laboratory data						
WBC, 10^9^/L (mean ± SD)	6.8 ± 2.6	6.5 ± 2.4	0.076	9.4 ± 4.6	9.1 ± 4.0	0.752
Hemoglobin, g/L (mean ± SD)	110.3 ± 19.0	122.4 ± 20.2	<0.001	110.9 ± 15.4	120.3 ± 20.1	0.001
Platelets, 10^9^/L (mean ± SD)	200.3 ± 62.3	171.0 ± 60.0	<0.001	212.7 ± 73.7	182.5 ± 64.2	0.009
Fasting glucose, mmol/l (median, IQR)	5.9 (4.9–6.9)	5.6 (4.8–6.8)	0.083	6.8 (4.9–8.2)	6.3 (5.3–7.7)	0.873
HbA1C, % (median, IQR)	6.5 (5.9–7.0)	6.2 (5.7–6.8)	0.115	7.0 (6.1–7.7)	5.8 (5.5–6.1)	0.035
ALT, u/l (median, IQR)	16.0 (10.9–22.9)	18.0 (12.4–27.8)	0.006	33.0 (20.7–48.5)	31.7 (16.7–58.8)	0.533
AST, u/l (median, IQR)	21.0 (16.2–26.9)	19.8 (16.9–27.2)	0.813	76.0 (25.7–286.4)	67.3 (28.7–187.7)	0.759
Albumin, g/L (mean ± SD)	35.7 ± 4.5	35.8 ± 3.8	0.785	32.9 ± 3.5	33.4 ± 4.1	0.494
Triglycerides, mmol/l (mean ± SD)	1.6 ± 1.1	1.7 ± 5.0	0.725	1.6 ± 0.8	1.3 ± 0.9	0.009
Total cholesterol, mmol/l (mean ± SD)	4.0 ± 1.0	3.7 ± 0.9	<0.001	4.4 ± 1.1	3.9 ± 0.9	0.002
HDL-cholesterol, mmol/l (mean ± SD)	1.1 ± 0.3	1.0 ± 0.3	0.002	1.1 ± 0.3	1.0 ± 0.3	0.373
LDL-cholesterol, mmol/l (mean ± SD)	2.3 ± 0.9	2.2 ± 0.8	0.018	2.7 ± 1.0	2.3 ± 0.8	0.005
Creatinine, *μ*mmol/l (mean ± SD)	91.3 ± 53.7	124.7 ± 109.1	<0.001	84.3 ± 56.0	123.4 ± 66.6	<0.001
Serum uric acid, *μ*mmol/l (mean ± SD)	352.6 ± 121.5	380.8 ± 106.9	0.005	336.6 ± 109.1	383.0 ± 118.1	0.013
PT, sec (mean ± SD)	13.7 ± 4.6	13.2 ± 3.0	0.367	13.6 ± 1.8	14.4 ± 1.7	0.164
APTT, sec (mean ± SD)	38.3 ± 10.4	39.4 ± 21.3	0.741	45.8 ± 27.5	47.9 ± 24.9	0.830
CK-Mb, u/l (mean ± SD)	19.6 ± 34.8	27.1 ± 91.3	0.280	91.0 ± 124.5	82.4 ± 158.2	0.725
TnT, pg/ml (median, IQR)	15.8 (8.9–49.1)	16.4 (10.8–66.4)	0.380	3092.5 (2437.8–4858.0)	2117.0 (1057.9–4761.0)	0.288
hs-CRP, mg/l (median, IQR)	2.7 (1.1–15.1)	3.4 (1.1–12.9)	0.813	24.5 (12.1–44.8)	14.9 (4.4–55.7)	0.328
NT-proBNP, pg/ml (median, IQR)	644.5 (225.5–2074.6)	755.3 (256.7–2273.2)	0.458	2751.7 (1418.2–6079.7)	3387.9 (1561.7–7932.8)	0.268
EF, % (mean ± SD)	55.3 ± 10.1	55.1 ± 10.1	0.862	47.9 ± 9.6	48.0 ± 11.5	0.961
In-hospital management						
Aspirin, no. (%)	174 (80.2)	261 (83.9)	0.267	53 (94.6)	115 (92.0)	0.524
Clopidogrel, no. (%)	183 (84.7)	273 (87.8)	0.312	51 (91.1)	118 (93.7)	0.533
ACEI/ARB, no. (%)	129 (59.4)	190 (60.9)	0.737	39 (69.6)	78 (61.9)	0.315
Beta blocker, no. (%)	167 (77.0)	218 (69.9)	0.072	39 (69.6)	88 (69.8)	0.979
Statin, no. (%)	210 (97.7)	298 (95.5)	0.191	55 (98.2)	124 (98.4)	0.923
Diuretic, no. (%)	93 (43.7)	124 (40.4)	0.457	35 (62.5)	86 (68.8)	0.405
PPI, no. (%)	173 (81.2)	223 (72.6)	0.024	54 (96.4)	117 (93.6)	0.441
IABP, no. (%)	1 (0.5)	8 (2.6)	0.089	6 (10.7)	10 (8.3)	0.609
Revascularization	49 (22.6)	114 (36.5)	0.002	33 (41.1)	60 (47.6)	0.159
In-hospital events						
Heart failure, no. (%)	25 (11.8)	29 (9.5)	0.404	17 (30.9)	48 (38.4)	0.335
Bleeding, no. (%)	14 (6.6)	17 (5.6)	0.618	8 (14.3)	22 (17.6)	0.579
Ventricular tachycardia, no. (%)	16 (7.5)	19 (6.2)	0.554	7 (12.5)	17 (13.6)	0.840
Stroke, no. (%)	3 (1.4)	1 (0.3)	0.310	2 (3.6)	1 (0.8)	0.177
Death, no. (%)	2 (2.2)	4 (3.0)	0.719	2 (8.7)	4 (8.7)	1.000
One-year follow-up						
Revascularization	4 (2.0)	6 (2.2)	0.891	0 (0.0)	1 (1.1)	0.480
Readmission, no. (%)	78 (37.3)	119 (40.5)	0.475	18 (35.3)	40 (38.5)	0.702
Stroke, no. (%)	10 (4.9)	16 (5.7)	0.698	3 (6.4)	2 (2.1)	0.193
Death, no. (%)	29 (13.4)	47 (15.1)	0.583	11 (19.6)	48 (38.1)	0.014

**Table 3:** Multivariable logistic regression analysis of one-year all-cause mortality.

	Unadjusted			Model 1			Model 2			Model 3		
	OR	95% CI	*p*	OR	95% CI	*p*	OR	95% CI	*p*	OR	95% CI	*p*
Total												
Women vs. men	0.62	0.41–0.93	0.021	0.60	0.40–0.90	0.014	0.53	0.35–0.81	0.003	0.57	0.35–0.91	0.018
Age	—	—	—	1.10	1.04–1.16	0.001	1.09	1.03–1.15	0.003	1.08	1.02–1.15	0.010
Severe heart failure	—	—	—	—	—	—	—	—	—	1.77	1.14–2.75	0.011
PLT	—	—	—	—	—	—	—	—	—	1.004	1.001–1.007	0.018
Creatinine	—	—	—	—	—	—	—	—	—	1.005	1.003–1.007	<0.001
STEMI												
Women vs. men	0.40	0.19–0.84	0.016	0.40	0.19–0.84	0.016	0.36	0.17–0.79	0.011	0.42	0.18–0.94	0.035
Severe heart failure	—	—	—	—	—	—	3.21	1.66–6.22	0.001	3.84	1.89–7.78	<0.001
NSTE-ACS												
Women vs. men	0.87	0.53–1.43	0.584	0.81	0.49–1.34	0.405	0.72	0.43–1.23	0.230	0.71	0.38–1.33	0.283
Age	—	—	—	1.13	1.05–1.20	<0.001	1.10	1.02–1.18	0.009	1.09	1.01–1.18	0.033
Severe heart failure	—	—	—	—	—	—	1.79	1.05–3.07	0.033	—	—	—
Hemoglobin	—	—	—	—	—	—	—	—	—	0.98	0.97–1.00	0.027
LDL	—	—	—	—	—	—	—	—	—	1.49	1.07–2.08	0.018
Creatinine	—	—	—	—	—	—	—	—	—	1.004	1.002–1.007	0.001
Serum uric acid	—	—	—	—	—	—	—	—	—	1.002	1.000–1.004	0.090

Data are expressed as OR ± 95% CIs (reported in parentheses) as assessed by univariate (unadjusted) or multivariate logistic regression analyses. Other covariates included in multivariate logistic regression models were as follows: Model 1: age; Model 2: age, diabetes mellitus, hypertension, current smoking, and severe heart failure; Model 3: adjustment for variables included age, diabetes mellitus, hypertension, current smoking, severe heart failure, hemoglobin, PLT, total cholesterol, HDL-C, LDL-C, creatinine level, and serum uric acid. The adjustment parameters which were statistically significant were shown.

## Data Availability

The data used to support the findings of this study are included within the Supplementary Materials.

## Supplementary Materials

Supplementary Materials A supplementary Excel datasheet file was used for statistical analysis.
